# The Amyloid Forming Peptides Islet Amyloid Polypeptide and Amyloid β Interact at the Molecular Level

**DOI:** 10.3390/ijms222011153

**Published:** 2021-10-15

**Authors:** Ye Wang, Gunilla T. Westermark

**Affiliations:** Department of Medical Cell Biology, Uppsala University, 75123 Uppsala, Sweden; Ye.wang@mcb.uu.se

**Keywords:** Aβ, BiFC, cross-seeding *Drosophila melanogaster*, IAPP

## Abstract

Epidemiological studies support a connection between the two common disorders, type-2 diabetes and Alzheimer’s disease. Both conditions have local amyloid formation in their pathogenesis, and cross-seeding between islet amyloid polypeptide (IAPP) and amyloid β (Aβ) could constitute the link. The bimolecular fluorescence complementation (BiFC) assay was used to investigate the occurrence of heterologous interactions between IAPP and Aβ and to compare the potential toxic effects of IAPP/Aβ, IAPP/IAPP, and Aβ/Aβ expression in living cells. Microscopy was used to confirm the fluorescence and determine the lysosomal, mitochondrial areas and mitochondrial membrane potential, and a FACS analysis was used to determine ROS production and the role for autophagy. *Drosophila melanogaster* expressing IAPP and Aβ was used to study their co-deposition and effects on longevity. We showed that the co-expression of IAPP and Aβ resulted in fluorophore reconstitution to the same extent as determined for homologous IAPP/IAPP or Aβ/Aβ expression. The BiFC(+)/BiFC(−) ratio of lysosomal area calculations increased in transfected cells independent of the vector combinations, while only Aβ/Aβ expression increased mitochondrial membrane potential. Expression combinations containing Aβ were necessary for the formation of a congophilic amyloid. In *Drosophila melanogaster* expressing IAPP/Aβ, co-deposition of the amyloid-forming peptides caused reduced longevity. The BiFC results confirmed a heterologous interaction between IAPP and Aβ, while co-deposits in the brain of *Drosophila* suggest mixed amyloid aggregates.

## 1. Introduction

Amyloid depositions are the primary pathological findings in a wide range of diseases, including type 2 diabetes (T2D) and Alzheimer’s disease (AD) [1]. The main amyloid constituent in T2D is islet amyloid polypeptide (IAPP), and amyloid deposits are present in the islet of Langerhans in more than 90% of the patients with the disease [2,3]. IAPP originates from the posttranslational processing of proIAPP [4,5], and the biologically active 37 residue-long polypeptide contains a disulfide bond between residues 2 and 7 and is C-terminally amidated. As a hormone, IAPP, in collaboration with insulin and glucagon, contributes to the maintenance of glucose homeostasis (reviewed in References [6,7,8]).

Peripheral insulin resistance and impaired hormone secretion from pancreatic beta cells are implicated in the pathogenesis of T2D [9]. Initially, peripheral insulin resistance can be compensated for by an enhanced secretion from the beta cells, accompanied by a simultaneous increase in prohormone secretion [10]. A high concentration of IAPP is an important but not decisive determinant for amyloid formation. Therefore, additional factors that trigger fibril formation are considered. Although IAPP makes up the main part of the amyloid, a small fraction of intracellular deposits contains proIAPP. In in vitro studies where proIAPP processing was impaired, the amyloid-forming propensity increased [11]. In cultured human islets and human IAPP-expressing transgenic mice islets, both membrane-enclosed and non-membrane-enclosed amyloid deposits developed in the cytoplasm [12].

In AD, a multifactorial neurodegenerative disease, amyloid deposits composed of misfolded amyloid beta (Aβ) are present in the cortex and blood vessels of the brain [13,14]. Aβ is generated from the amyloid precursor protein (AβPP) by sequential cleavage with β- and γ-secretases and occurs with several different fragment lengths [15]. The Aβ 1–40 fragment is most dominant in AD, while Aβ 1–42 is the dominant fragment in deposits in the nondemented elderly population [16,17]. AβPP, which is a cell surface receptor with an essential role in neuronal growth and synapse formation [18,19], is widely expressed together with necessary convertases in various tissues. However, Aβ amyloid is primarily limited to the central nervous system, where the amyloid deposits occur mainly extracellularly. Still, the identification of intracellular proteins in the amyloid deposit suggests a pathway that includes an intracellular route [20].

Monomeric IAPP and Aβ are both natively unfolded peptides [21], and a comparison of IAPP and Aβ amino acid sequences reveals an overall 25% amino acid sequence identity. This unusually high degree of similarity between the two unrelated amyloid peptides is even higher for the regions involved in fibril formation. This resemblance was early recognized [22], and O’Nuallain et al. [23] used the ThT assay to study heterologous seeding between IAPP and Aβ and showed that the addition of preformed Aβ 1–40 and Aβ 1–42 aggregates to a monomeric IAPP solution triggers IAPP fibrillation comparable to that observed for homologous IAPP seeding. Binding studies of IAPP and Aβ identified two IAPP regions (IAPP 8–18 and IAPP 22–28) with s high binding affinity for Aβ. The latter IAPP region, residues 22–28, contains the GAILS sequence essential for IAPP self-aggregation [24]. Additionally, in the same binding study, three regions on Aβ (Aβ 19–22, Aβ 27–32, and Aβ 35–40) were identified to have a high affinity for IAPP. The three identified Aβ regions are also important for the self-assembly of Aβ.

In an in vivo seeding experiment, a single intravenous injection of preformed IAPP fibrils to human IAPP transgenic mice (hIAPP) resulted in an almost 10-fold increase in IAPP amyloid-containing islets compared to mice injected with the vehicle [25]. In a heterologous seeding experiment where preformed Aβ fibrils were used as the seed, a single intravenous injection to hIAPP transgenic mice resulted in a five-fold increase in IAPP amyloid-containing islets. This experiment indicated that cross-seeding occurred, but it was not possible to determine whether fibril extended through the addition of monomers onto the ends of the Aβ seed or whether the propagation was a result of secondary nucleation. The proximity ligation assay (PLA), a highly sensitive and specific antibody-based technology, generated a positive signal on AD brain sections when IAPP and Aβ antibodies were combined [25,26]. However, in this system, the presence of IAPP does not ensure that the peptides bind to each other.

Bimolecular fluorescence complementation (BiFC) is a technique suitable for the visualization of protein–protein interactions in living cells [27]. The two proteins of interest are fused with either the N-terminal or C-terminal fragment of a fluorescent protein, respectively. When expressed in cells, two interactive proteins bring together the two nonfluorescent fragments to reconstitute the fluorescent protein, which can be detected using fluorescence microscopy. The detection range of the BiFC assay is approximately 6–10 nm [28].

## 2. Results

### 2.1. Aβ and IAPP Interact on a Molecular Level

**Figure 1:**
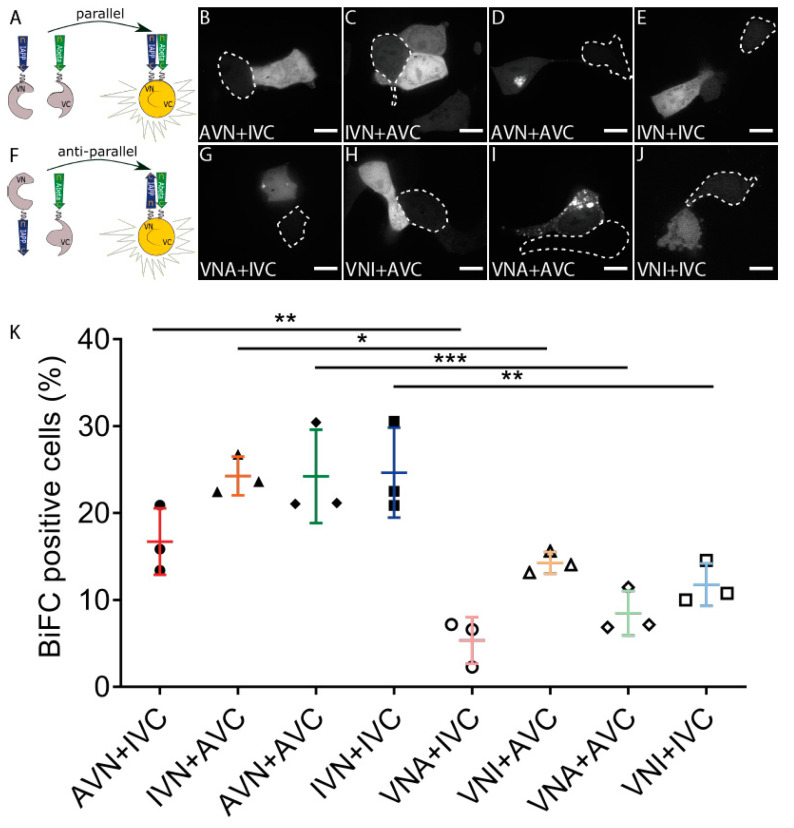
Aβ and IAPP prefer parallel binding over antiparallel binding in living HEK293 cells. (**A**) Schematic drawing of parallel binding in BiFC. Confocal images of BiFC-positive cells with the parallel binding of Aβ and IAPP in (**B**) AVN + IVC, (**C**) IVN + AVC, (**D**) AVN + AVC, and (**E**) IVN + IVC. (**F**) Schematic drawing of antiparallel binding in BiFC. Confocal images of BiFC-positive cells with antiparallel binding of Aβ and IAPP in (**G**) VNA + IVC, (**H**) VNI + AVC, (**I**) VNA + AVC, and (**J**) VNI + IVC. Dotted circles denote BiFC-negative cells. (**K**) Proportions of BiFC-positive cells in both parallel and antiparallel settings were determined by flow cytometry. Scale bars: 10 µm. Results were analysed by one-way ANOVA with Bonferroni’s post hoc analysis and presented as the mean ± SD, * *p* < 0.05, ** *p* < 0.01, and *** *p* < 0.001, *n* = 3.

We used the BiFC technique and studied the molecular interaction between IAPP and Aβ E22G (Figure 1A). The three-letter code used to specify the vectors describes the order of the three parts, and the definition of the abbreviations is as follows: I for IAPP, A for Aβ, V for Venus, and N and C define the N-terminal fragment residues 1–173 and C-terminal fragment residues 155–239, respectively. The fluorescent signal observed in cells transfected with AVN + IVC, IVN + AVC, AVN + AVC, or IVN + IVC supports peptide binding, followed by a subsequent assembly of Venus (Figure 1B–E), a diffuse cytoplasmic fluorescence developed independently of the construct composition. Together with the transfected cells in Figure 1, nontransfected cells are present and identified by a dotted line for comparison. To investigate whether peptide orientation affected the binding, we produced vectors with the different parts inserted in reversed order (Figure 1F) (VNA and VNI). The co-transfection of VNA + IVC, VNI + AVC, VNA + AVC, and VNI + IVC generated a fluorescence similar to that observed for peptides aligned in a parallel direction (Figure 1G–J). In some Aβ-expressing cells, diffuse cytoplasmic fluorescence was accompanied by a well-defined dot-like fluorescence (Figure 1D,G,I).

The FACS analysis used to quantify the transfection efficiency showed that almost 25% of the cells transfected with IVN + AVC, AVN + AVC, and IVN + IVC and 17% of the cells transfected with AVN + IVC exhibited a fluorescent signal. In comparison, transfection with VNA + IVC, VNI + AVC, VNA + AVC, and VNI + IVC resulted in 5%, 12%, 8%, and 10% fluorescent cells, respectively (Figure 1K). Furthermore, a comparison of counterparts, e.g., AVN + IVC and VNA + IVC, showed that peptides expressed in a parallel orientation exhibit a higher degree of peptide binding than peptides expressed in an antiparallel orientation. The results suggest that the peptides interact preferably when in a parallel orientation.

### 2.2. In TEM, Dot-Like Inclusion Bodies Contain a Mixture of Amorphous and Fibrillar Material

HEK293 cells transfected with AVN + AVC and IVN + IVC were fixed with 2.5% glutaraldehyde and processed for TEM with cells still attached to the cell culture dish. This procedure was used, since trypsin treatment makes adherent cells round up during detachment, and morphological change may cause displacement of the intracellular components.

**Figure 2:**
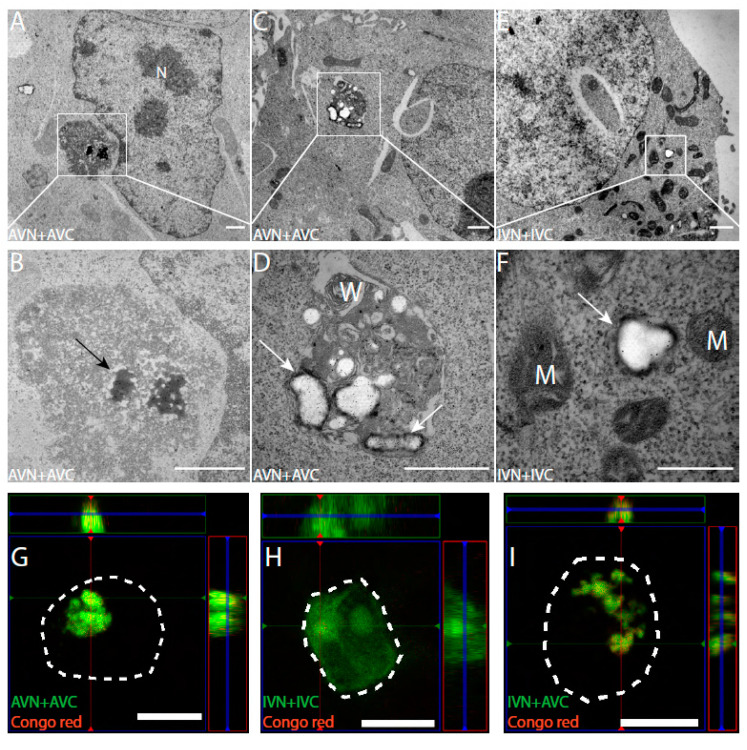
Transfection with AVN + AVC results in electron-dense fibrillary aggregates with an affinity for Congo red. The immuno-TEM analysis of HEK293 cells transfected with (**A**–**D**) AVN + AVC and (**E**,**F**) IVN + IVC. (**A**,**B**) Electron-dense aggregates with Aβ immunoreactivity (black arrow) located adjacent to the cell nucleus. (**C**,**D**) Amorphous structures with Aβ immunoreactivity (white arrows) located close to membranous whorls. (**E**,**F**) Diffused IAPP immunoreactivity (white arrow) is detected in the cytoplasm. Orthogonal images from z-stack showing BiFC signal and Congo red staining in transfected HEK293 cells. Colocalization between BiFC and Congo red signals is observed in cells transfected with (**G**) AVN + AVC and (**I**) IVN + AVC but not in (**H**) cells transfected with IVN + IVC. The dashed lines denote the boundaries of cells. M: mitochondria, N: nucleus, and W: membranous whorls. Scale bars: 1 µm (**A**–**E**), 500 nm (**F**), and 10 µm (**G**–**I**).

Ultrathin sections placed on Ni grids were immunolabelled with anti-Aβ and anti-IAPP antibodies to identify expressed proteins. In AVN + AVC-transfected cells, as expected, there was diffuse cytoplasmic labeling with anti-Aβ antibodies. In the same cells, abundant labeling was observed in electron-dense areas present in the cytoplasm but adjacent to the cell nucleus (Figure 2A,B). Both fibrillar and amorphous components were present in the aggregates, which lack membrane enclosure. The location and size of several micrometers in diameter suggest that they correspond to the fluorescent dot-like aggregates observed in the confocal microscope. In some cells transfected with AVN + AVC, an accumulation of membrane structures containing amorphous materials was recognized by anti-Aβ antibodies (Figure 2C,D). Cells transfected with IVN + IVC showed a diffuse cytoplasmic immunoreactivity, and no amorphous or fibrillar aggregates were present. In other areas, condensed mitochondria with dense matrix components and wide crista could be seen (Figure 2E,F).

### 2.3. Amyloid Develops in Cells Expressing Aβ-Containing BiFC Constructs

Twenty-four hours after transfection with AVN + AVC and IVN + IVC, HEK 293 cells were stained for amyloid with Congo red. In cells transfected with AVN + AVC, Congo red fluorescence coincided with the dot-like fluorescence described above (Figure 2G). However, no Congo red fluorescence appeared in cells transfected with IVN + IVC, and these cells did not contain a dot-like Venus fluorescence (Figure 2H). Additionally, after the heterologous transfection (IVN + AVC), Congophilic dot-like deposits appeared (Figure 2I).

### 2.4. Aβ/IAPP, Aβ/Aβ, and IAPP/IAPP Expression Increase Lysosomal and Mitochondrial Area, but Only Aβ/Aβ Expression Elevates MMP

**Figure 3:**
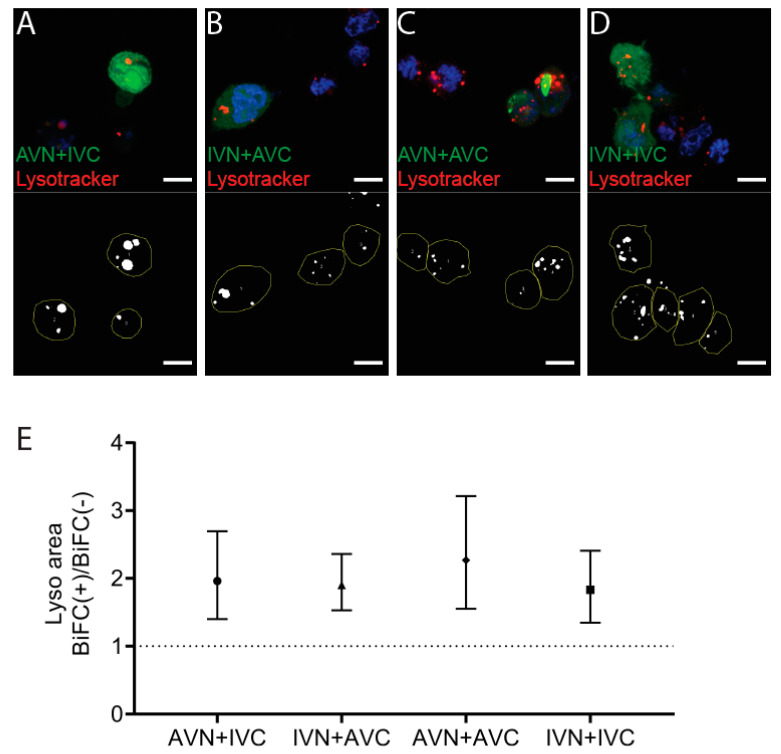
Expression of Aβ, IAPP, and Aβ + IAPP increase the lysosomal area. Confocal images of HEK293 cells transfected with (**A**) AVN + IVC, (**B**) IVN + AVC, (**C**) AVN + AVC, and (**D**) IVN + IVC stained with Lysotracker (red) and DAPI (blue). Below are the corresponding binary images displaying the cell area (yellow circle) and lysosomal area (white). (**E**) The ratio of the lysosomal area in BiFC(+)/BiFC(−) cells of AVN + IVC, IVN + AVC, AVN + AVC, and IVN + IVC-transfected cells. Scale bar: 10 µm. The results were analyzed by the method of EC Fieller and presented as the mean with a 95% confidence interval.

The accumulation of dot-like perinuclear inclusion bodies present in cells expressing Aβ/Aβ or the combination of IAPP/Aβ may result from reduced clearance. After transfection, the cells were incubated with the weak basic ligand Lysotracker, and the lysosomal area per cell area in BiFC-positive cells was compared to the lysosomal area determined in the nonfluorescent control cells (Figure 3A–D). In AVN + IVC, the mean lysosomal area in BiFC-positive cells was 7.4%, compared to 3.8% in control cells (*p* < 0.001). In IVN + AVC, the mean lysosomal area in BiFC-positive cells was 9.8% compared to 5.2% in the control cells (*p* < 0.0001). In AVN + AVC, the mean lysosomal area in BiFC-positive cells was 9.7% compared to 4.3% in the control cells (*p* < 0.001), while, in IVN + IVC, the mean lysosomal area in BiFC-positive cells was 9.1% compared to 5.0% in the control cells (*p* < 0.01). For comparison, the ratio of BiFC(+)/BiFC(−) are presented (Figure 3E), and independent of the vector combinations used for the transfection, the lysosomal area in BiFC-positive cells was increased. However, comparing the lysosomal area between the four BiFC-positive cell groups showed no significant difference. Therefore, it is unlikely that the dot-like fluorescence arises as a result of deteriorating degradation.

**Figure 4:**
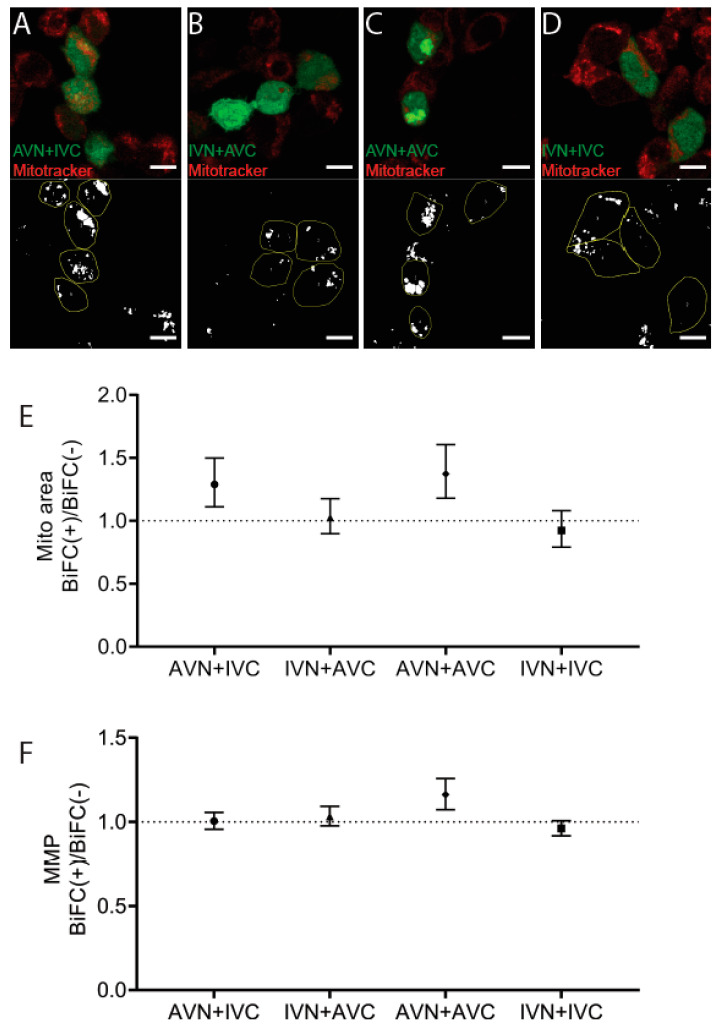
The expression of Aβ/Aβ increases the mitochondrial membrane potential. Confocal images of HEK293 cells transfected with (**A**) AVN + IVC, (**B**) IVN + AVC, (**C**) AVN + AVC, and (**D**) IVN + IVC stained with mitotracker (red). Below are the corresponding binary images displaying the cell area (yellow circle) and mitochondrial area (white). (**E**) The ratio of the mitochondrial area in BiFC(+)/BiFC(−) cells of AVN + IVC, IVN + AVC, AVN + AVC, and IVN + IVC-transfected cells. (**F**) The ratio of MMP in BiFC(+)/BiFC(−) cells of AVN + IVC, IVN + AVC, AVN + AVC, and IVN + IVC-transfected cells. Scale bar: 10 µm. Results were analyzed by the method of EC Fieller and presented as a mean with a 95% confidence interval.

Mitotracker was used to identify mitochondria 48 h post-transfection with AVN + IVC, IVN + AVC, AVN + AVC, and IVN + IVC (Figure 4A–D). This cationic probe containing a thiol-reactive chloromethyl moiety enters functional mitochondria and becomes fluorescent upon oxidization. The mitochondrial area per cell area increased in BiFC-positive cells transfected with AVN + IVC to 8.8%, compared to 6.8% in the control cells (*p* < 0.001) and after transfection with IVN + AVC, the area increased to 10% in BiFC-positive cells compared to 9.8% in untransfected cells (ns). Following transfection, with AVN + AVC, the area increased to 10.4% in BiFC-positive cells compared to 7.6% in the control cells (*p* < 0.0001). After transfection with IVN + IVC, the mitochondrial area was determined up to 9.1% in BiFC-positive cells and to 9.9% in the control cells (ns). When comparing the BiFC(+)/BiFC(−) ratios (Figure 4E), an increase in the mitochondrial area occurred after transfection with AVN + IVC and AVN + AVC, while, in cells transfected with IVN + AVC, the mitochondrial area was unchanged. In cells transfected with IVN + IVC, a slight decrease in the mitochondrial area of BiFC-positive cells was observed.

Since an increase in the mitochondrial area does not necessarily result in an increased function, the fluorescence intensity, a measure of the mitochondrial membrane potential (MMP) and reflecting mitochondrial activity, was determined. The comparison of the BiFC(+)/BiFC(−) ratio (Figure 4F) showed that it was only in BiFC-positive cells transfected with AVN + AVC that an increase in MMP was observed compared to non-transfected cells. The results suggest that, in cells expressing Aβ/Aβ, an increase in the mitochondrial area was accompanied by the rise in MMP. In cells transfected with AVN + IVC, the increased mitochondrial area ratio was comparable to that determined for cells transfected with Aβ/Aβ. However, this increase did not affect the MMP levels. Correspondingly, in cells transfected with IVN + AVC and IVN + IVC, the mitochondrial area did not change, and the MMP remained unaffected (Figure 4F). Flow cytometry was used to replicate the mitotracker analysis, and these results were consistent with those obtained from the confocal microscopy analysis (data not shown).

### 2.5. Expression of Aβ, IAPP, and Aβ/IAPP Increase Superoxide Production and Susceptibility to Cell Death

**Figure 5:**
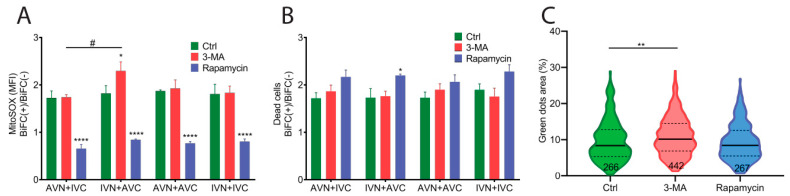
Expression of Aβ, IAPP, and Aβ + IAPP led to a higher superoxide production and induced cell death. (**A**) The ratio of mitochondrial superoxide production detected by the mean fluorescence intensity (MFI) of mitoSOX in BiFC(+)/BiFC(−) cells of AVN + IVC, IVN + AVC, AVN + AVC and IVN + IVC-transfected cells in the control group, 3-MA-treated group, and rapamycin-treated group. (**B**) The ratio of PI-positive dead cells in BiFC(+)/BiFC(−) cells of AVN + IVC, IVN + AVC, AVN + AVC, and IVN + IVC-transfected cells in the control group, 3-MA-treated group, and rapamycin-treated group. (**C**) Violin plot shows the fluorescent dots area (%) in AVN + AVC-transfected cells at 48 h post-transfection. Cells were treated with 3-MA or rapamycin at 6 h and 24 h post-transfection. (**A**,**B**) The results were analyzed by two-way ANOVA with Bonferroni’s multiple comparison test and presented as the mean ± SEM; * *p* < 0.05, **** *p* < 0.0001, and # *p* < 0.05, *n* = 3. (**C**), The results were analyzed by one-way ANOVA with Dunnett’s multiple comparison test; ** *p* < 0.01.

We examined the stress response in BiFC-positive cells using mitoSOX to determine the superoxide levels in mitochondria 48 h after transfection. Transfection with IVN + AVC, AVN + IVC, AVN + AVC, and IVN + IVC resulted in higher superoxide production in BiFC-positive cells than the control cells. The data presented as BiFC(+)/BiFC(−) shows no difference between the vector combinations (Figure 5A).

The accumulation of intracellular protein aggregates has been shown to activate autophagy [29,30]. To investigate if autophagy was activated by BiFC expression, transfected cells were cultured in the presence of the autophagy inhibitor 3-methyladenine (3-MA) and the autophagy inducer rapamycin. The cells were analyzed 48 h after transfection. In cells exposed to 3-MA, the BiFC(+)/BiFC(−) ratio was similar to control cells regardless of the constructs used in the transfection (Figure 5A). It was only in cells transfected with IVN + AVC that the determined ROS production was significantly higher than in cells transfected with AVN + IVC. The upregulation of autophagy by rapamycin in transfected cells eliminated the difference in superoxide production between BiFC-positive cells and control cells (Figure 5A).

In parallel with the MitoSOX quantifications, PI was used to analyze the cell viability 48 h after transfection. Elevated cell death was observed in BiFC-positive cells compared to untransfected control cells. After transfection, cell death in BiFC-positive and control cells was determined to be 60% and 40%, respectively. The results are shown as a BiFC(+)/BiFC(−) ratio (Figure 5B). In transfected cells cultured in the presence of the autophagy inhibitor 3-MA, the measured cell death was comparable to that obtained from untreated transfected cells (Figure 5B). Rapamycin reduced the overall cell death compared to untreated transfected cells and 3-MA treated cells, but it was only in IVN + AVC-transfected cells that an increase in the BiFC(+)/BiFC(−) ratio was observed (Figure 5B).

The fluorescent dot area was quantified in cells transfected with AVN + AVC and cultured in a medium supplemented with 3-MA or rapamycin. The inhibition of autophagy with 3-MA increased the size of the fluorescent inclusion bodies, while potentiation of the autophagy with rapamycin did not affect the clearance of the fibrillar aggregates (Figure 5C).

### 2.6. Co-Expression of Aβ and IAPP Shortens the Lifespan in Drosophila melanogaster

**Figure 6:**
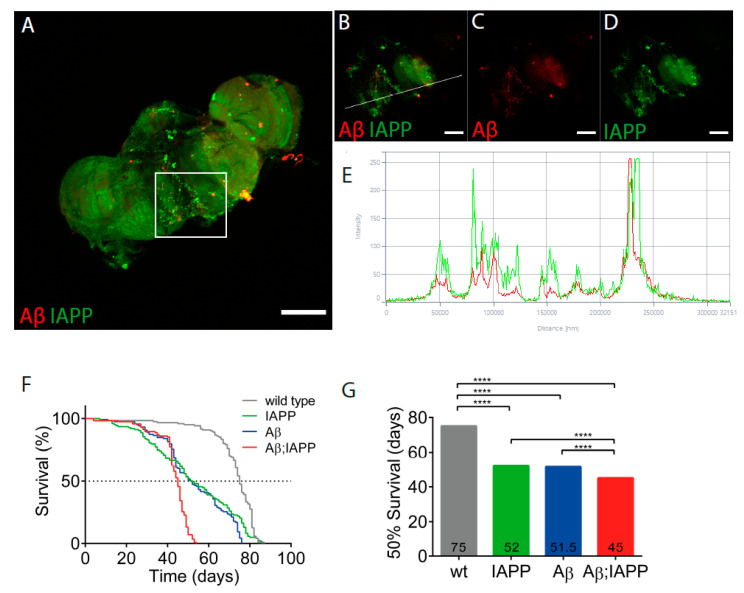
Co-expression of IAPP and Aβ has exaggerated the toxic effects on *Drosophila’s* lifespan. (**A**) Confocal image of a 10-day-old *Drosophila* brain recovered from a fly with Aβ + IAPP expression driven by elavC155 and immunolabeled with anti-Aβ antibody (red) and anti-IAPP antibody (green). (**B**–**D**) Insets of (**A**). (**E**) Fluorescence profile of Aβ (red) and IAPP (green) displaying colocalization in (**B**), as indicated by the white line. (**F**) The fraction of surviving flies over time was plotted for control flies (grey), flies expressing IAPP (green), flies expressing Aβ (blue), and flies expressing Aβ + IAPP (red). (**G**) Median survival times were calculated for control flies and flies expressing IAPP, Aβ, and Aβ + IAPP. Comparison between curves were made using the log-rank (Mantel–Cox) test; **** *p* < 0.0001. Scale bar: 50 µm.

*Drosophila melanogaster* expressing human IAPP, Aβ, or Aβ;IAPP, driven by the pan-neuronal driver elavC155-Gal4, were used in the co-deposition studies. In brains dissected from 10-day-old double-transgenic flies, immunostaining for IAPP and Aβ revealed deposits labeled with both antibodies, which is indicative of the colocation of the two peptides (Figure 6A–D), a finding confirmed by the fluorescence profile (Figure 6E). Additionally, in brains from double-transgenic flies, single peptide deposits were also present. Only the expected immunoreactivity appeared in the brains of single transgenic flies expressing Aβ or IAPP (data not shown).

As a measure of the toxicity, the survival of flies expressing Aβ, IAPP, and Aβ + IAPP, driven by elavC155-Gal4, was determined. Female flies used for the analysis were kept in groups of 25 flies per vial at 26 °C. For the control, female offspring from w1118 flies crossed with elavC155-Gal4. Double-transgenic flies expressing Aβ + IAPP had a median lifespan of 45 days, a significantly shorter lifespan than the control flies with a median lifespan of 75 days (*p* < 0.0001). The lifespan for flies expressing Aβ or IAPP alone had a median lifespan of 52 days (Figure 6F,G).

## 3. Discussion

The BiFC technology was introduced almost two decades ago and has since then undergone several improvements. We selected Venus for complementation; this bright yellow fluorophore is stable at pH 7.6–8.0 [31], and the split was positioned to generate the N-terminal fragment residues 1–173 and C-terminal fragment residues 155–238. Separation of the molecule at these positions results in an overlap that, after refolding, stabilizes the three-dimensional structure of Venus [32]. In the present study, Aβ E22G 42, also known as Aβ arctic, was used for IAPP interaction studies. The APP mutation E693G increases the β secretase cleavage of the precursor, leading to an increase in protofibril formation [33]. Since the mutation at position 22 appears outside the IAPP-binding sequences Aβ 27–32 and Aβ 35–42 identified by Andreetto et al. [24], it is not expected to interfere with the IAPP and Aβ interaction that occurred in the HEK293 cells after transfection. In the BiFC systems, a linker is often used to connect the protein of interest and the nonfluorescent fragment. Linkers with different lengths have been described [34], and in a pilot study, we tested both five (RSIAT) and 17 (RPACKIPNDLKQKVMNH) amino acid residue long linkers. Complementation of the fluorophore were monitored, and under the used experimental condition, the transfection of cells with BiFC constructs containing the RSIAT linker did not give rise to any fluorescent signal (not shown), indicating that the short linker restrained the flexibility and did not allow for complementation of the two nonfluorescent N-terminal and C-terminal fragments. However, in cells transfected with BiFC constructs with a 17 amino acid linker, a fluorescence signal appeared, and this was used.

BiFC was used to confirm the formation of heterodimers between the local amyloid-associated peptides IAPP and Aβ when co-expressed in cells. We used IAPP and Aβ homologous interactions for comparison, and fluorescent cells were confirmed with microscopy and FACS analyses. Almost 25% of the cells transfected with IVN + AVC showed fluorescence, and this was at the same level obtained for cells transfected with IVN + IVC or AVN + AVC. The result supports the emergence of heterologous interaction in vivo, at an efficacy comparable to the homologous interaction. Cells transfected with the AVN + IVC combination exhibited a slightly reduced number of fluorescent cells (Figure 1K), but the mRNA analysis revealed comparable expression levels for the different constructs, and availability should not be a limiting factor (Figure S1). In the BiFC assay, the peptide interaction must be the determining step and drive the creation of the fluorophore. To ensure that this was the situation under the used conditions, a competition analysis was performed where cells were transfected with an equal concentration of BiFC vectors and a non-fluorophore-coupled peptide. The principal behind this was that binding of a non-fluorophore-linked peptide to any of the coupled peptides prevented further interactions and thereby inhibited the generation a functioning fluorophore. Competition with uncoupled IAPP or Aβ resulted in a 20–25% reduction in fluorescent cells (Figure S2). We did not quantify the intensity of the fluorescent signal; instead, the number of cells was determined, and the obtained decrease in cell number points to effective prevention of fluorophore restoration by the co-expression of unlinked peptides.

There are few publications on BiFC analysis performed on amyloid-associated peptide interactions. In 2008, Outerio and collaborators used BiFC and studied the formation of cytotoxic oligomers from a-synuclein (a-Syn) [35]. They used GFP as a reporter and analyzed a variety of BiFC vector combinations. A weak fluorescent signal was observed when a-Syn was expressed in a parallel direction (aSyn-GN and aSyn-GC), while they obtained a strong fluorescent signal after transfection with a-Syn in an antiparallel orientation (GN-link-aSyn and aSYN-GC). To determine in which orientation IAPP and Aβ bound to each other or themselves, we produced BiFC vectors where the fluorescent fragment was placed in front of the peptide and where antiparallel interaction was required for the fluorophore to reconstitute. After the transfection of cells with vector combinations for antiparallel interactions, a number of fluorescent cells were reduced, and we concluded that all four tested combinations preferred a parallel orientation instead of an antiparallel direction.

An interesting observation was the development of inclusion bodies in cells transfected with the BiFC constructs containing Aβ. Most inclusion bodies were present in cells transfected with the BiFC vector combinations AVN + AVC or VNA + AVC (Figure 1). The electron microscopy examination and Congo red staining showed that fluorescent inclusion bodies consisted of Aβ in fibrillar and amorphous forms. Although to a lesser extent, inclusion bodies appeared in cells transfected with any combination aimed for heterologous expression (AVN + IVC, IVN + AVC, VNA + IVC, and VNI + AVC). The colocalization of Congo red fluorescence and the Venus signal originating from IAPP + Aβ interactions supports the fibrillation of mixed fibrils (Figure 2I). Earlier, we showed that preformed Aβ fibrils can seed IAPP in vivo [25], but the obtained results from the BiFC expression supports the formation of heterologous interactions between IAPP and Aβ amyloid fibrils. Besides dimerization studies of a-Syn, BiFC has been used to investigate the oligomerization and assembly of higher-order aggregates [36,37]. Still, there are no reports on the amyloid formation [38], as described herein. The difference may depend on the protein expressed.

IAPP resides a high propensity to form amyloid and in the commonly used ThT assay that allows monitoring of the amyloid fibril formation; the peptide usually displays a short lag phase, while the Aβ peptide lag phase is usually significantly longer. Therefore, it was a bit surprising that homologous expression of IAPP did not give rise to inclusion bodies or any fibrillary materials.

The detected fibrillar aggregates resembled, partly, the Aβ aggregates described by Bückig et al. [39], as they were nonmembrane-enclosed and located in the perinuclear area. In contrast to their aggregates, which were also found in the nucleus, the inclusion bodies described herein were limited to the cytosol. Misfolded proteins escaping proteasomal degradation can be actively transported to the aggresome [40] for storage or future degradation by selective autophagy, and the packing of misfolded proteins into the aggresome is recognized to be cytoprotective [41]. Therefore, the accumulation of inclusion bodies could be a sign of reduced clearance. However, the lysosomal area was increased in all fluorescent cells compared to the control cells, and this was independent of the BiFC construct used and not limited to constructs causing inclusion bodies. The observed increase in the mitochondrial area detected in AVN + AVC-transfected cells and, to a lesser extent, in cells transfected with the heterologous combination AVN + IVC indicates an increased ATP production, but the mitochondrial membrane potential increased only in cells transfected with AVN + AVC. Despite the increase, ATP production may remain insufficient. ROS production was elevated in fluorescent cells independent of the BiFC combinations used for transfection. However, there is no difference in cell death between the groups of transfected cells. Autophagy is suggested to be responsible for the removal of the aggresome. This was shown to be true for aggresome formed during the expression of aggregation-prone proteins, such as mutant Huntingtin and Tau [42]. 3-MA prevents the formation of the autophagosome and inhibits clearance by autophagy. The addition of 3-MA to transfected cells resulted in a general increase in ROS production both in BiFC-positive and BiFC-negative cells, but this did not result in an increase in cell death. Instead, the BiFC(+)/BiFC(−) cell death ratio remained comparable to the ratio determined for untreated cells. Autophagy inhibition increased the inclusion body area in cells transfected with the AVN + AVC. However, something that argues against autophagy as a significant pathway for the degradation of the inclusion bodies is that a culture in the presence of rapamycin did not reduce the inclusion body area. All in all, the findings argue against autophagy being responsible for degradation of the inclusion bodies, and it is possible that their formation prolongs survival but that the accumulation eventually leads to cell death.

Transgenic *Drosophila melanogaster* provides an important model for protein misfolding diseases [43,44]. The expression of human IAPP, Aβ E22G, and the combination of IAPP + Aβ E22G all reduce the survival (Figure 6), and the expression of IAPP + Aβ E22G was more toxic than IAPP or Aβ E22G expression alone. IAPP and Aβ E22G are expressed behind a signal peptide, and both are expected to be secreted from the neurons, and from the images depicted, the immune reactivity appears to be extracellularly. In the double-transgenic flies, we could observe three different deposits composed of IAPP/Aβ, IAPP, and Aβ (Figure 6E). The intensity profile reveals the colocalization and supports amyloid deposits composed of IAPP and Aβ. Recently, Bharadwaj et al. [45] allowed IAPP and Aβ to form hetero-oligomers in vitro, and the morphological analysis identified aggregates with morphology distinct from homologous oligomers. Cytotoxicity studies on SH-SY5Y cells showed that co-aggregates of Aβ/IAPP were more toxic to the cells than the addition of aggregates composed of IAPP or Aβ alone. One possible explanation for the observed increased cytotoxicity could be the increased ability for IAPP/Aβ aggregates to interact with cell membranes [46].

## 4. Materials and Methods

### 4.1. Material Used for the Study

Human Embryonic Kidney 293 (HEK293) cells (ATCC CRL-1573TM) were purchased from ATCC, VA, USA. Oligonucleotides and primers were synthesized at TAG Copenhagen, Denmark, and the Aβ antibody (6E10) was from Covance (Princeton, NJ, USA). The pcDNA3.1 zeo + vector, Alexa-labeled secondary antibodies, were from Invitrogen (Waltham, MA, USA). Restriction enzymes, Turbofect, DAPI, Lysotracker (L7528), Mitotracker (M7513), MitoSOX (M36008), Fetal Bovine Serum (FBS), Penicillin–Streptomycin, Trypsin, and sodium pyruvate were from Thermo Fisher Scientific (Waltham, MA, USA). RPMI 1640 medium, β-mercaptoethanol, Congo red, 3-Methyladenine (M9281), rapamycin (R8781), propidium iodide (P4170), and regular salts for buffers were from Sigma-Aldrich (St. Louis, MO, USA). Epon was from Electron Microscopy Science (Hatfield, PA, USA), and disposable materials for the cell culture were from Corning (Corning, NY, USA).

### 4.2. BiFC Vectors

All vectors contained three components: protein of interest, linker, and N-terminal or C-terminal part of fluorescent protein Venus. Abeta E22G 42 and human IAPP were amplified using PCR primer pairs containing HindIII or EcoRI restriction sites (Table 1). A 17-amino acid long linker (RPACKIPNDLKQKVMNH) with restriction sites (EcoRI and XbaI) was synthesized as the oligonucleotides. The selected linker sequence described by Hu CD et al. [27,32] enabled mobility between the peptide of interest and the Venus fragment. The oligonucleotides were denatured at 95 °C for 5 min and were allowed to anneal while the solution cooled down to room temperature. The splitting sites of the N-terminal and C-terminal Venus were chosen at residue 173 and 155, respectively, as described by Hu CD et al. [47]. N-terminal and C-terminal Venus parts were produced with PCR using primer pairs containing XbaI or ApaI restriction sites. All fragments were, after digestion with the corresponding restriction enzymes, cloned into a pcDNA3.1 zeo+ vector. The expected sequence of the insert was confirmed by sequencing (Eurofins, Germany).

### 4.3. Cell Culture and Transfection

HEK293 cells were maintained in RPMI 1640 medium supplemented with 10% FBS, 1% Penicillin–Streptomycin, 1% sodium pyruvate, and 0.05-mM β-mercaptoethanol at 37 °C with 5% CO_2_. Cells were split every other day. For transfection, 50,000 cells were seeded per well in a 24-well plate the day before transfection. One microgram of DNA was mixed with 2-µL Turbofect and added to the cells.

### 4.4. Congo Red Staining

Transfected cells grown on coverslips were incubated with Congo red B solution (80% ethanol saturated with NaCl and Congo red and a final concentration of 0.01% NaOH) for 1 min and rinsed with absolute ethanol for 30 s. Cells were mounted with 8-mM Na2HPO4, 2-mM KH2PO4, 137-mM NaCl, and 2.7-mM KCl pH7.4 (PBS) glycerol (1:1); sealed with nail polish; and imaged directly using a confocal microscope.

### 4.5. Transmission Electron Microscopy

Twenty-four hours post-transfection, cells were fixed in 2.5% glutaraldehyde and embedded in Epon. Ultra-thin sections were placed on formvar-coated nickel grids, blocked with 3% BSA in 50-mM tris buffer with 150-mM NaCl pH 7.4 (TBS) for 30 min, and incubated with primary antibody (A133 or 6E10) overnight. The next day, sections were washed 3 × 5 min in TBS and blocked with 3% BSA in TBS for 30 min before incubated with a proper secondary antibody labeled with 10-nm gold particles for 1 h. Both primary and secondary antibodies were diluted with 1% BSA in TBS at 1:200 dilution. After rinsing with TBS and water, specimens were contrasted with 2% uranyl acetate in 50% ethanol for 10 min and Reynold solution (120-mM sodium citrate and 25-mM lead citrate) for 3 min before viewed using Hitachi H-7100 transmission electron microscope (Hitachi, Tokyo, Japan). Images were captured using Gatan multiscan camera with Gatan digital micrograph software version 3.6.4 (Gatan, Pleasanton, CA, USA).

### 4.6. Detection of Lysosomes

Forty-eight hours post-transfection, cells grown on coverslips were incubated with 50-nM Lysotracker for 30 min at 37 °C. Cells were rinsed in PBS and fixed in 4% paraformaldehyde (PFA) for 15 min. Both steps were carried out at 37 °C. Subsequently, cells were rinsed in PBS, mounted with DAPI in PBS glycerol (1:1), and stored at 4 °C until imaged. The area of lysosomes was quantified using FIJI software [48]. BiFC-positive cells and BiFC-negative cells were manually identified, and the cell area was determined. Thereafter, the lysosomal areas were measured using auto-threshold MaxEntropy, and the result was presented as the lysosomal area/cell area in %.

### 4.7. Detection of Mitochondria

Forty-eight hours post-transfection, cells grown on coverslips were incubated with 500-nM Mitotracker for 30 min at 37 °C. Cells were rinsed in lukewarm PBS and fixed in 4% paraformaldehyde (PFA) for 15 min. Subsequently, cells were rinsed in PBS, mounted with DAPI in PBS glycerol (1:1), and stored at 4 °C until imaged. Area and the fluorescence intensity of mitochondria were analyzed using FIJI software. BiFC-positive cells and BiFC-negative cells were manually identified, and the cell area was determined. Mitochondria were measured using auto-threshold MaxEntropy. The mitochondria areas were measured individually and displayed as the total cell mitochondrial area/cell area (%). Additionally, the mean fluorescence intensity of each mitochondrial area was calculated and expressed as MMP.

### 4.8. Detection of Mitochondrial Superoxide

Forty-eight hours post-transfection, cells were trypsinized at 37 °C for 5 min and collected. Cells were centrifuged at 1000 rcf for 5 min, the supernatant was removed, and cells were incubated with 1-µM MitoSOX for 30 min at 37 °C to label mitochondrial superoxide. Cells were subsequently centrifuged at 1000 rcf for 5 min, and the supernatant containing unbound MitoSOX was removed. The pellet was resuspended in PBS, and cells were analyzed using an Accuri C6 plus flow cytometry machine (BD Biosciences, NJ, USA). Cells were compensated for Venus and MitoSOX signals before being gated for living cells and singlets. The gating of BiFC-positive cells was determined using MitoSOX-stained untransfected HEK 293 cells. The mean fluorescence intensity of MitoSOX was determined after background correction using cells supplemented with an equal volume of DMSO as used for the solubilization of MitoSOX (Figure S3).

### 4.9. Modulation of Autophagy

Inhibition of autophagy was achieved using 3-Methyladenine (3-MA)) on transfected HEK293 cells. A series of concentrations ranging from 0.1 mM to 10 mM were tested, and a final concentration at 5 mM was selected for our experiments. 3-MA was solubilized in a preheated cell culture medium and filtered before added to the cells at time points 6 h and 24 h post-transfection. Cells were analyzed at 48 h post-transfection.

Induction of autophagy was achieved with rapamycin on transfected HEK 293 cells. A series of concentrations ranging from 10 nM to 5 µM were examined, and a final concentration of 100 nM was chosen for our experiments. Rapamycin-containing medium was added to the cells at time points 6 h and 24 h post-transfection. Cells were analyzed at 48 h post-transfection.

### 4.10. Detection of Dead Cells

Forty-eight hours post-transfection, the cell culture medium was recovered to collect any floating cells. The attached cells were trypsinized at 37 °C for 5 min and mixed with the aforementioned cell culture medium. Cells were then centrifuged at 1000 rcf for 5 min, and the pellet was resuspended in ice-cold PBS with 2-µg/mL propidium iodide (PI) and analyzed using flow cytometry. Cells were compensated for Venus and PI before gated using untransfected HEK293 cells. The proportion of dead cells was determined for BiFC-positive and BiFC-negative cells, respectively.

### 4.11. Confocal Imaging

Live-cell images were taken twenty-four hours after transfection using Yokogawa VSU-10 spinning disc confocal system attached to Eclipse TE-2000 microscope (Nikon, Tokyo, Japan). Images for mitochondria and lysosomes, as well as *Drosophila* brains, were taken using Zeiss LSM 780 laser scanning confocal microscope (Zeiss, Oberkochen, Germany). Images for Congo red staining were taken using Zeiss LSM 700 laser scanning microscope (Zeiss). All images were analyzed using Zen software (Zeiss) and Fiji software [48].

### 4.12. Drosophila melanogaster

Transgenic flies expressing human IAPP (UAS-IAPP) [5], Aβ E22G (UAS-Aβ) [6], and a double transgenic expressing IAPP and Aβ E22G (UAS-IAPP-Aβ) were used. The transgene expression was driven by the Gal4-elavC155 driver line obtained from Bloomington *Drosophila* stock center, Indiana University. The Gal4-elavC155 driven expression results in a pan-neuronal expression pattern of the transgenes. Female virgins from each transgenic line were crossed with Gal4-elav C155 males. The longevity analysis was performed on mated female offspring collected 24 h after hatching. Each sample group consisted of one hundred flies kept in groups of 25 flies on standard food at 26 °C. Food was changed every 2 or 3 days, and the number of dead flies was determined daily.

Fly brains were dissected in PBT (PBS + 0.3% Triton-X), fixed in 4% PFA for 20 min, rinsed with PBT 3 × 20 min, and blocked in PBT supplemented with 5% FBS for 30 min at room temperature. Primary antibodies targeting IAPP (A133, raised in rabbit against human IAPP residues 20–29) and Aβ (6E10) were diluted 1:100 in blocking buffer. Fly brains were incubated with primary antibodies for 3 days at 4 °C. Subsequently, fly brains were rinsed 3 × 20 min in PBT and further incubated with secondary antibodies (Alexa goat-anti-rabbit 488 and Alexa donkey-anti-mouse 555, both diluted 1:200 in blocking buffer) for 4 days at 4 °C. After rinses 3 × 20 min in PBT, fly brains were mounted with PBS glycerol and imaged.

### 4.13. Statistics

All statistical analyses were performed on GraphPad Prism 8 (GraphPad Software, San Diego, CA, USA). Unpaired Student’s *t*-test was used to compare two groups, while one-way ANOVA with Bonferroni’s post hoc analysis or Dunnett’s post hoc analysis was used to analyze studies with more than two groups. Fiellers’s theorem [49] was used for calculation of a 95% confidence interval for the ratio of two means. Log-ranked Mantel-Cox test was used for comparing survival curves of *Drosophila*.

## 5. Conclusions

We used BiFC to study the interaction between the amyloid-associated peptides IAPP and Aβ. The result shows that co-expression of IAPP and Aβ results in a comparable number of fluorescent cells as detected after transfection with Aβ/Aβ or IAPP/IAPP, implying the efficient formation of heterologous interactions. Fluorescent inclusion bodies frequently formed in cells transfected with Aβ/Aβ, and to a lesser extent, in cells transfected with Aβ/IAPP. Congo red staining reveals the presence of amyloid. In line with this are deposits in the brain of double transgenic expressing flies recognized by anti-IAPP and anti-Aβ antibodies. The results imply that IAPP and Aβ form heterologous interactions that can propagate to amyloid fibrils in living cells.

## Figures and Tables

**Table 1:** PCR primers used for BiFC constructs.

IAPP	Forward: 5′-TATTAAGCTTATGAAATGCAACACT-3′ Reverse: 5′-AAGGGAATTCATATGTATTGGATCC-3′
Aβ	Forward: 5′-TATTAAGCTTATGGATGCGGAATTT-3′ Reverse: 5′-AAGGGAATTCCGCAATCACCAC-3′
Venus 1-173 (VN):	Forward: 5′-TTAATCTAGAGTGAGCAAGGGC-3′ Reverse: 5′-AAACGGGCCCCTATTACTCGATGTTGTG-3′
Venus 155-239 (VC)	Forward: 5′-TTAATCTAGAGCCGACAAGCAG-3′ Reverse: 5′-AAACGGGCCCCTATTACTTGTACAGCTC-3′
Venus 1-173 (VN) Antiparallel	Forward: 5′-TTAAAAGCTTATGGTGAGCAAGGGC-3′ Reverse: 5′-AAACGAATTCCTCGATGTTGTG-3′
IAPP Antiparallel	Forward: 5′-TATTTCTAGAAAATGCAACACT-3′ Reverse: 5′-AAGGGGGCCCCTATTAATATGTATTGGA-3′
Aβ Antiparallel	Forward: 5′-TATTTCTAGAGATGCGGAATTT-3′ Reverse: 5′-AAGGGGGCCCCTATTACGCAATCACCAC-3′

## Supplementary Materials

The following are available online at https://www.mdpi.com/article/10.3390/ijms222011153/s1: Figure S1. A similar expression level of the BiFC vectors was reached in HEK293 cells 24 h after transfection. Figure S2. Interaction between the peptides IAPP, Aβ, or the combination of IAPP and Aβ drives the recreation of Venus. Figure S3. Gating strategy for MitoSOX staining.

## Abbreviations

Alzheimer’s disease, AD; amyloid beta, Aβ; amyloid beta E22G, Aβ E22G; amyloid precursor protein, AβPP; bimolecular fluorescence complementation, BiFC; islet amyloid polypeptide, IAPP; MMP, mitochondrial membrane potential; and type 2 diabetes, T2D.
