# Supplementary figures and images for: The Amyloid Forming Peptides Islet Amyloid Polypeptide and Amyloid β Interact at the Molecular Level
Source: Int J Mol Sci. 2021 Oct 15;22(20):11153. doi: 10.3390/ijms222011153 (PMC8541034; doi:10.3390/ijms222011153)

**A**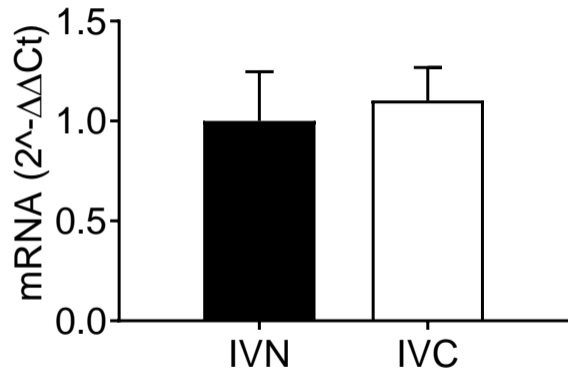**B**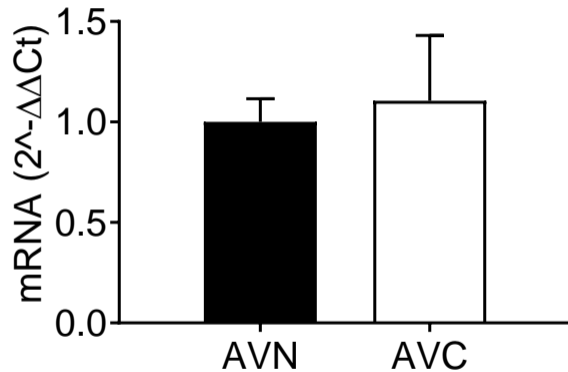

Supplement: Supplementary file 1 [file ijms-22-11153-s001.zip › Figure S1, Wang Y. et al.pdf]

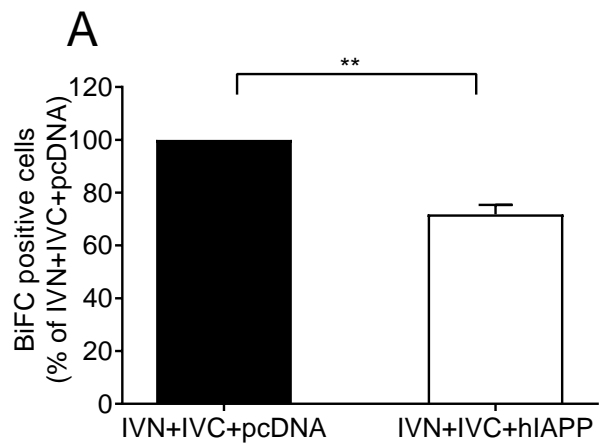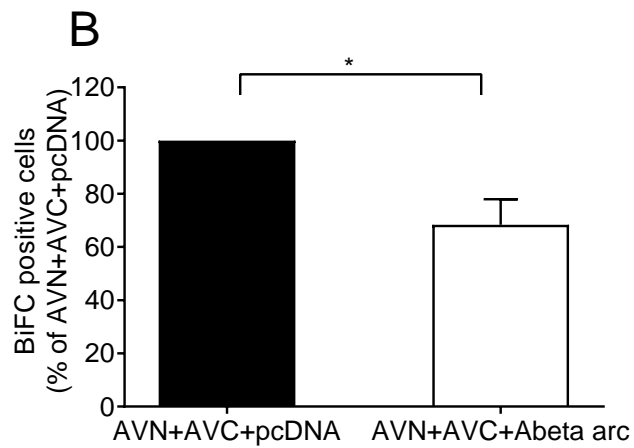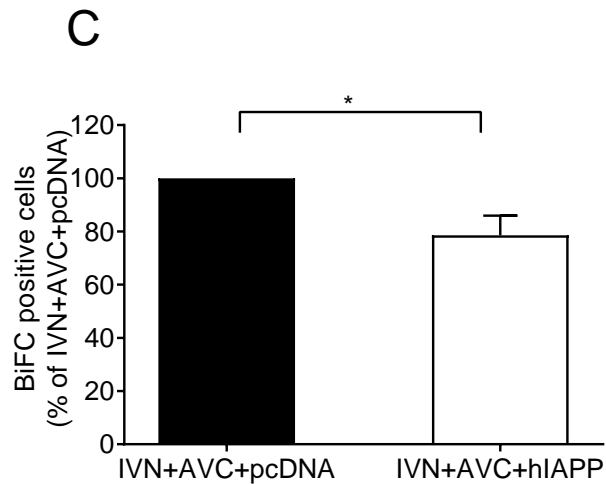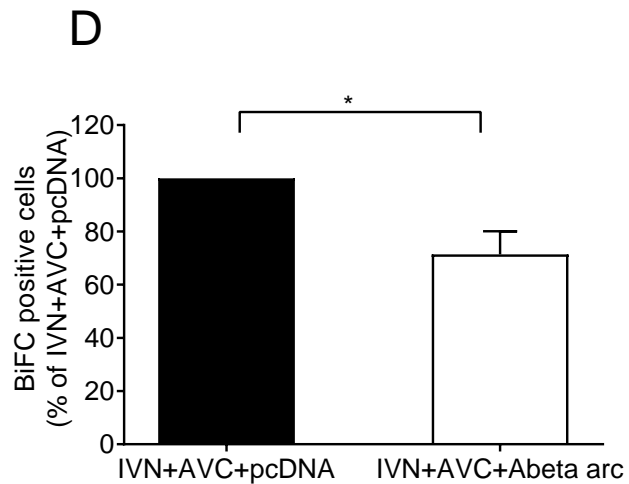

Supplement: Supplementary file 1 [file ijms-22-11153-s001.zip › Figure S2, Wang Y. et al.pdf]

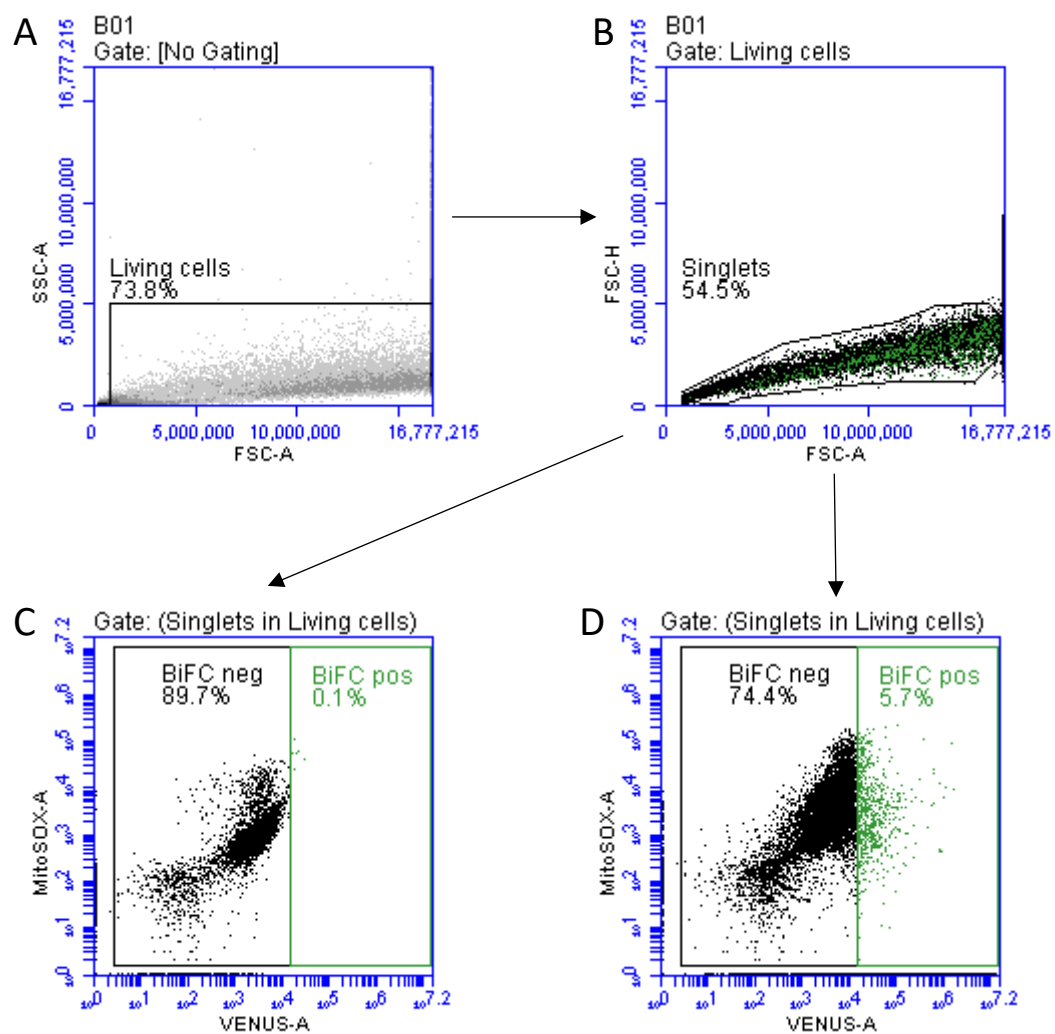

Supplement: Supplementary file 1 [file ijms-22-11153-s001.zip › Figure S3, Wang Y. et al.pdf]
